# Young Adult With Malignant Phyllodes Undergoing Adjuvant Radiotherapy With Bolus

**DOI:** 10.1002/ccr3.73471

**Published:** 2026-09-28

**Authors:** Dominic Rafie, Mickie Baca, Stephanie Rice

**Affiliations:** ^1^ Department of Radiation Oncology Northern Arizona Healthcare Flagstaff Arizona USA

**Keywords:** adjuvant radiotherapy, breast neoplasms, mastectomy, radiation dosage

## Abstract

Malignant phyllodes tumor of the breast (MPTB) is a rare fibroepithelial tumor and very uncommon among young adult women. It has a higher likelihood of local recurrence (LR) compared to other phyllodes tumors of the breast. Due to limited data, there is no established consensus regarding adjuvant radiotherapy for MPTB. Post‐Mastectomy Radiotherapy (PMRT) reduces the risk of LR and breast cancer mortality. A bolus can be used to treat LR by counteracting the skin‐sparing effect, as well as in cases where body habitus or tissue expander location may require a bolus to deliver the full dose to the region. Since MPTB is aggressive and therefore delivery of the full dose is needed, a bolus may be beneficial for patients with MPTB. This case report is, to our knowledge, the first to mention a young adult with MPTB undergoing adjuvant radiotherapy with a bolus. A 23‐year‐old white female presented with a fibroepithelial neoplasm in her right lateral breast with no history of breast surgery, biopsy, or radiation and no skin changes, nipple discharge, or inversion. The patient underwent a skin‐ and nipple‐sparing simple mastectomy of the right breast due to residual MPTB from the prior lumpectomy. She received 5,000 cGy in 25 fractions (19 fractions without bolus and 6 fractions with 0.5 cm bolus) to the right chest wall, followed by a 1,000 cGy boost in 5 fractions using Volumetric Modulated Arc Therapy. A bolus was used for 6 of the fractions since it was difficult to give the full dose to her close margin due to the tissue expander making a portion of the concerning region flat on the chest wall. Radiotherapy was well‐tolerated, with side effects of soreness, itchiness, Grade 2 radiation dermatitis, skin hyperpigmentation, dry desquamation, and fatigue as side effects. Therefore, due to the risk of LR, her high‐risk factors, potential risk of metastasis, and difficulties to deliver full dose due to body habitus and tissue expander location, this patient benefitted from adjuvant radiotherapy using a bolus. Furthermore, this case report adds to the scarce literature of young adults with MPTB, particularly on the use of a bolus for adjuvant radiotherapy.

**Key Clinical Message**

Post‐Mastectomy Radiotherapy with a bolus may be used for malignant phyllodes tumor of the breast in patients with difficult tissue expander location or body habitus to provide dose to the chest wall region and to reduce the risk of local recurrence in female breast cancer patients with high‐risk features.

## 1 Introduction

Primary neoplasms are unusual in young adult women, with most being benign [1]. Phyllodes tumor of the breast (PTB) is a palpable, rapidly growing mass that makes up approximately 0.3%–0.5% of all primary female breast neoplasms, with an incidence of 2.1 per million women (approximately 500 cases per year) in the US, and is usually found in women aged 35–55 years old [1, 2, 3, 4, 5, 6, 7]. According to the World Health Organization, the three variants of PTB are benign, borderline, and malignant, with the differentiation of each based on cellular atypia, infiltrative margins, and mitotic frequency [8].

Malignant phyllodes tumor of the breast (MPTB) is a rare fibroepithelial tumor, consisting of progressive epithelial and mesenchymal components that originate from the periductal and intralobular stroma [6]. It predominantly occurs in middle‐aged and elderly women and constitutes 6.5%–27% of all PTB [1, 2, 9, 10]. It is not only rarer to see in younger patients, but also potentially a sign of worse prognosis, as some studies also suggest that age is a significant predictor of MPTB prognosis [11]. Relative to other PTBs, MPTB has a higher propensity for local recurrence (LR) and distant metastasis in the lungs, with the former being associated with an increased risk of death and the latter leading to a dismal prognosis [2, 3, 12, 13, 14, 15, 16, 17].

Historically, there has not been an established consensus regarding the use of adjuvant radiotherapy for MPTB, due to limited data [4, 7, 14, 17, 18]. Some studies have reported the successful use of adjuvant radiotherapy for margin‐positive post‐mastectomy patients [3]. Post‐Mastectomy Radiotherapy (PMRT) has been shown to reduce the risk of LR and breast cancer mortality in several retrospective studies and one prospective study [13, 18, 19]. During PMRT, a bolus can be used to counteract the skin‐sparing effect, as well as in cases where body habitus or tissue expander location may require a bolus to deliver the full dose to the region to treat the LR [19]. Since MPTB is more aggressive and has a higher rate of LR than other forms of PTB, using a bolus may be beneficial for patients with MPTB [1, 7]. Here we present a case report that, to our knowledge, is the first to feature a young adult with MPTB undergoing adjuvant radiotherapy while using a bolus.

## 2 Case Presentation/Examination

A 23‐year‐old white female initially self‐palpated an abnormality in her right lateral breast. She sought medical attention at our institution after this mass began to enlarge and she started to experience pain in Fall 2023. Upon physical examination, bilateral breasts were mildly asymmetric (*R*>L) with grade A ptosis. Her large mass comprised the entire lateral right breast (UOQ), with otherwise no skin changes, nipple discharge, or nipple inversion. She is premenopausal (G0P0) and has no history of breast surgery, biopsy, or radiation.

Diagnostic ultrasound of the right breast confirmed a suspicious abnormality. Of concern was a large heterogeneous, predominantly hypoechoic mass vs. conglomerate of masses. Ultrasound‐directed biopsy was recommended, which found a fibroepithelial neoplasm measuring 6.8 cm × 2.9 cm × 6.4 cm at the 9 to 10 o'clock position 3 cm from the nipple in the right breast. There was no suspicious abnormality in the right axilla.

The breast surgeon performed a right breast lumpectomy (10.0 × 5.8 × 5.2 cm) and ensured minimal external incisions as the patient keloids significantly. Excision of the tumor provided evidence that was most consistent with primary MPTB, with atypical spindle cell proliferation within the right inferior and medial margins of the right breast (positive margin). Histology and pathology further confirmed the finding of MPTB, as mitotic activity was 28 mitoses per 10 high‐power fields (HPF). The tumor had stromal expansion without definitive overgrowth and increased stromal cellularity with mild to moderate atypia. Oriented excision of the left breast was performed, exhibiting fibrocystic mastopathy with no atypia or carcinoma. A Chest CT with contrast showed no acute abnormalities and no findings of metastatic disease.

Genetic testing was ordered, which indicated variants of uncertain significance (VUS) in PMS2 (c.1477G>A, p.Asp493Asn), POLE (c.461G>A, p.Arg154Lys), SDHA (c.985C>G, p.Arg329Gly), and SMARCA4 (c.4297G>A, p.Glu1433Lys). Family history consisted of her maternal grandfather with renal cell carcinoma, paternal grandmother with hepatocellular carcinoma, maternal uncle with head and neck squamous cell carcinoma, and a paternal uncle with bladder cancer.

## 3 Differential Diagnosis, Investigations, and Treatment

The patient underwent a skin‐ and nipple‐sparing simple mastectomy of the right breast due to residual MPTB involving the region of the prior lumpectomy, which spanned 7.0 cm with a close margin measuring 0.25 mm posteriorly. The resection margin of the simple mastectomy was very close posteriorly (0.25 mm). A prepectoral tissue expander was placed immediately after mastectomy. No dermal invasion was reported.

She had a consultation regarding adjuvant chemotherapy, which was not recommended by medical oncology. The patient was then evaluated and recommended for adjuvant radiotherapy to reduce (LR) risk. After a detailed risk/benefit discussion, she consented to adjuvant radiation to her right chest wall and proceeded to a CT simulation of the right chest wall. She has no pacemaker or previous radiotherapy, and her urine b‐HCG was negative. Using Volumetric Modulated Arc Therapy (VMAT), the patient received 5,000 cGy in 25 fractions (19 fractions without bolus and 6 fractions with bolus) to the right chest wall, followed by a 1,000 cGy boost in 5 fractions to the right central and upper medial quadrants of her chest wall and her mastectomy scar line (Table 1 and Figure 1). A 0.5 cm bolus with a specific gravity of 1.02 from Radiation Products Design was used. The dosimetric data of the organs at risk and coverage to the right chest wall and scar boost are depicted in Table 2. All plans were created by a certified medical dosimetrist and reviewed by the radiation oncologist and qualified medical physicist. All constraints were met and approved by the radiation oncologist.

**TABLE 1:** Overview of adjuvant radiotherapy treatment regimen.

Plan	Energy	Fractions	Dose per Fraction (cGy)	Dose Correction (cGy)	Total Dose (cGy)	Elapsed Days
Rt CW VMAT	6X	19/19	200	0	3800	34
Rt CW VMAT Bolus	6X	6/6	200	0	1200	14
Rt SCAR Boost	6X	5/5	200	0	1000	6
Plan Sum					6000	

Abbreviations: Bolus = 0.5 cm; CW = Chest Wall; Rt = Right; VMAT = Volumetric Modulated Arc Therapy.

**FIGURE 1:**
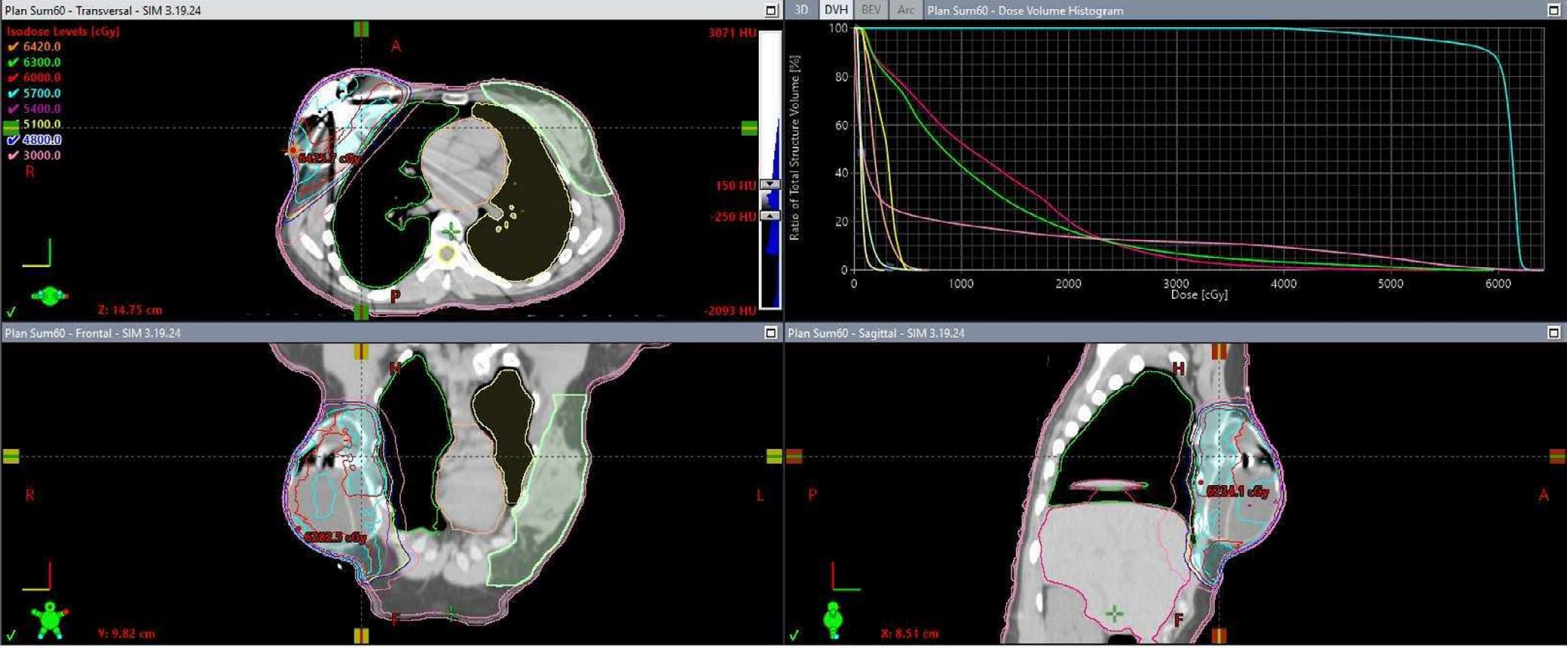
Plan Sum of Rt CW VMAT, Rt CW VMAT with Bolus, and Rt SCAR Boost treatment plans. Total dose is 60 Gy. Dose‐Volume Histogram in the top right contains the following structures: PTV60 = Cyan, Breast_L = Light Green, Heart = Peach, Liver = Hot Pink, Lung_L = Light Yellow, Lung_R = Green, Skin = Pink, Spinal Cord = Yellow.

**TABLE 2:** Dosimetric data of right chest wall and scar boost.

Plan ID	RT Lung V20 (%)	Heart Dmean (cGy)	Skin of Chest Wall D95% (cGy)
Rt CW VMAT	2.5	98.7	
Rt CW VMAT Bolus	2.1	84.0	
Rt SCAR Boost	0.0	23.9	
Plan Sum (6,000 cGy)	16.5	206.6	3748.5

*Note:* The skin of the Chest Wall is defined as a 0.3 cm inner wall margin subtraction from the body contour along the Chest Wall.

Abbreviations: Bolus = 0.5 cm, CW = Chest Wall, Rt = Right, VMAT = Volumetric Modulated Arc Therapy.

## 4 Conclusion and Results

PTB is a rare neoplasm of breast cancer, with MPTB being an uncommon form of PTB that is typically not found among young adults [1, 20, 21]. Due to the risk of LR and the low but potential risk of metastasis, it is pertinent to thoroughly treat MPTB with adjuvant radiotherapy using a bolus, especially when the patient has high‐risk factors and has a tissue expander that does not allow for the close margin to be treated with full dose without a bolus [15]. Therefore, this case report adds to the scarce literature of young adults with MPTB, particularly as a use case for when a bolus may be used for adjuvant radiotherapy.

The patient tolerated radiotherapy well, with side effects of soreness, itchiness, Grade 2 radiation dermatitis, and skin hyperpigmentation, dry but no wet desquamation, and fatigue. Trental and Vitamin E were prescribed to prevent radiation fibrosis but were discontinued early due to nausea from Trental. She has healed well and underwent implant exchange with placement of a contralateral smaller implant for symmetry with plastic surgery. She remains active and working, with a good range of motion. She continues to do physical therapy to proactively break up scar tissue and reduce any lymphedema risk. At her latest follow‐up, which was twelve months after the initial abnormality was discovered, she was Signatera negative and has no evidence of disease.

## 5 Discussion

MPTB is higher among white and East Asian females, with the former aligning with the identity of our patient [15, 22]. She reported initial pain at the tumor site, which is atypical as most patients report an enlarging painless tumor mass [22, 23]. She found her mass while performing a self‐examination in Fall 2023, which is in line with previous studies that mention the most common presentation of PTB being a visible, palpable mass [10, 22].

It was determined that the patient had a form of PTB and not a fibroadenoma because, while both neoplasms are composed of epithelial and connective tissue, her tumor had higher stromal cellularity, which is mentioned in the literature as a differentiating factor [7]. While Lohitvisate and colleagues [10] found that menopausal status significantly increases the possibility of a non‐benign PTB, Boutrus and colleagues [4] found no statistically significant difference between benign and non‐benign menstrual status. This discrepancy may be due to sample size differences, as Lohitvisate and colleagues [10] had a sample size of 57 patients compared to the 108 patients of Boutrus and colleagues [4]. In our case, since the patient is 23 years old, she is premenopausal.

In terms of tumor features, almost every criterion of MPTB was met except for stromal overgrowth [20]. According to Ultrasonography, her MPTB was hypoechoic, which has been previously reported for PTB [10]. The location of her tumor was entirely within the UOQ, which, as previously reported in other studies, is the most frequent location for PTB [10, 22]. This patient had no nodal metastasis or metastases in general, which is in line with previous studies suggesting that PTBs in general have little metastatic potential [7, 22]. Since all four (PMS2, POLE, SDHA, and SMARCA4) of the genetic mutations the patient had were VUS, they could not be considered relevant for clinical decision‐making [24]. However, it is interesting to note that the loss of SDHA function caused by a mutation is observed in renal cell carcinoma, which is the same cancer her maternal grandfather had [12].

A simple mastectomy was performed rather than wide local excision or lumpectomy alone due to the tumor being large and because the histology confirmed the tumor as MPTB, which requires mastectomy as the standard surgical approach [10, 14]. As reported by Reinfuss and colleagues [5], simple mastectomy was the most common operation for MPTB. Adjuvant chemotherapy was not given as there is little evidence to suggest it is a useful modality for this patient [22, 25]. Adjuvant radiotherapy was performed on this patient due to having a tumor size larger than 5 cm, greater than 10 mitoses per 10 HPF, and having positive margins after lumpectomy [7, 26]. Our patient had close margins postoperatively, which was the reason to recommend a boost to the quadrant where the close margin was located. Therefore, adjuvant radiotherapy was also given as it aligns with previous studies that have seen success in treating patients with close or positive margins post‐mastectomy [3].

Dahn and colleagues [19] found in their systematic review that while there is a lack of high‐quality literature regarding the use of PMRT with a bolus, for tumors with high‐risk features such as skin involvement and positive margins, a bolus may be recommended to achieve dose to superficial regions and reduce the risk of LR. While this tumor had no skin involvement or positive margin, the location of the close margin made it difficult to ensure full dose, with little distance between the skin and the chest wall/pectoralis muscle. Despite being located posteriorly, it was in the upper medial portion of the right chest wall. The tissue expander spanned a portion of this, but a portion of the concerning area was flat on the chest wall, and to obtain dose to this region, the use of a bolus was required. When a bolus was not used, full dose was unable to be obtained in this region; hence the need for coverage with the use of a bolus due to body habitus and location of the tissue expander. Therefore, since this patient had MPTB, with features predictive of recurrence such as positive and very close margin status after the lumpectomy and mastectomy respectively, stromal atypia, higher mitotic activity, and a tissue expander and body habitus which made full dose treatment to the close margin difficult to achieve sans bolus, a bolus was used to obtain adequate dose to the superficial chest wall [13, 18]. A disadvantage of using a 0.5 cm bolus daily is that it is significantly associated with developing Grade 3 radiation dermatitis. Since the patient only received 6 out of 30 fractions with the bolus, we reduced the risk of radiation dermatitis. A bolus was used on our patient, who has a tissue expander, as there is no significant difference in risk of wet desquamation or Grade 3 radiation dermatitis between use of bolus or no bolus. A 0.5 cm bolus was used in lieu of a 1.0 cm as thicker boluses tend to lead to increased toxicity when used daily.

## Author Contributions

**Dominic Rafie:** conceptualization, data curation, investigation, writing – original draft, writing – review and editing. **Mickie Baca:** data curation, writing – review and editing. **Stephanie Rice:** data curation, supervision, writing – review and editing.

## Funding

The authors have nothing to report.

## Disclosure

This case report was written at Cancer Centers of Northern Arizona Healthcare—Flagstaff.

## Ethics Statement

As case reports are not considered human research, no local ethics committee approval was needed for this case report. Written informed consent was obtained from the patient for publication. All patient information and data were anonymized, with no HIPAA identifiers present within the case report.

## Consent

The authors obtained written informed consent from the patient for use of medical information to be published online.

## Conflicts of Interest

The authors declare no conflicts of interest.

## Data Availability

The data that support the findings of this study are available on request from the corresponding author. The data are not publicly available due to privacy or ethical restrictions.
